# Utility of Follow-Up Surveillance Echocardiograms in Uncomplicated Surgical Closures of Perimembranous Ventricular Septal Defects: A Preliminary Analysis

**DOI:** 10.3390/jcdd13060281

**Published:** 2026-06-22

**Authors:** Macala Maney, Carson Richardson, Isaac Kistler, Samantha Fichtner, Hannah Jacobs, Julie B. Aldrich, Clifford L. Cua

**Affiliations:** 1 The Heart Center, Nationwide Children’s Hospital, Columbus, OH 43205, USA; macala.maney@nationwidechildrens.org (M.M.); samantha.fichtner@nationwidechildrens.org (S.F.); hannah.jacobs@nationwidechildrens.org (H.J.); julie.aldrich@nationwidechildrens.org (J.B.A.); 2 Center for Biostatistics, College of Medicine, The Ohio State University Wexner Medical Center, Columbus, OH 43210, USA; carson.richardson@nationwidechildrens.org (C.R.); isaac.kistler@nationwidechildrens.org (I.K.); 3 Biostatistics Resource at Nationwide Children’s Hospital, Abigail Wexner Research Institute at Nationwide Children’s Hospital, Columbus, OH 43205, USA

**Keywords:** perimembranous VSD, surgical closure, follow-up echocardiogram, ventricular septal defect

## Abstract

**Background**: Ventricular septal defects (VSD) are the most common form of congenital heart disease (CHD). Current guidelines recommend surveillance transthoracic echocardiograms (TTE) following surgical closure of perimembranous VSDs (pVSD); however, duration of screening is not explicitly stated. The goal of this study is to determine the utility of follow-up TTEs after uncomplicated pVSD surgical closure. **Methods**: Single-site retrospective analysis was conducted on patients who underwent pVSD surgical closure. Patients were excluded if diagnosed with other CHD, had complications 1 year post-repair, or lacked data 1 year post-repair. Serial TTEs were reviewed. A Kaplan–Meier curve was used to illustrate the 5-year complication-free survival. **Results**: A total of 117 patients met inclusion criteria. A 97% 5-year complication-free survival was observed. Four patients had complications >1 year post-repair: one non-obstructive subaortic ridge, one pulmonary vein stenosis, one pinhole residual pVSD, and one ventricular ectopy with ventricular dysfunction. Of the 113 complication-free patients, 197 TTEs were performed with no change in clinical management. **Conclusions**: Beyond 1 year post-repair, the occurrence of new complications following uncomplicated pVSD surgical closure is rare. Unless clinical concerns arise, the utility of routine TTEs > 1 year post-repair in this uncomplicated post-surgical cohort should be reassessed. Larger multicenter studies are needed to determine the utility of routine TTEs.

## 1. Introduction

Ventricular septal defects (VSD) are the most common form of congenital heart disease (CHD), comprising 40–50%, and are found to be isolated lesions in about 20% of patients [1,2,3]. The incidence of a VSD is approximately 2–4 per 1000 live births, with perimembranous VSD (pVSD) being the most prevalent type [1,2,4]. Of the estimated 15,000 patients born with VSDs, approximately 20% undergo surgical closure annually with excellent outcomes and few complications as improvements in surgical techniques and cardiopulmonary bypass are made [4,5].

For patients with CHD, there are established guidelines for the appropriate use of imaging for surveillance screening as well as monitoring for complications following repair. These recommendations were established as a collaborative effort among numerous societies including the American College of Cardiology, American Heart Association, American Society of Echocardiography, and the Society of Pediatric Echocardiography, with guidance from expert opinion and existing clinical trial data [6]. Current guidelines recommend obtaining follow-up transthoracic echocardiograms (TTE) within the first 30 days following surgical VSD closure as well as within 1 year if the patient is asymptomatic or has only mild sequela. Subsequent surveillance TTEs are recommended every 2–3 years; however, the duration of continued surveillance TTEs is not explicitly stated [6].

The central objective of this study is to determine the utility of routine surveillance TTEs following uncomplicated surgical closure of a pVSD through identification and description of post-operative complications occurring beyond 1-year post-repair as well as timing of those complications.

## 2. Materials and Methods

### 2.1. Study Population and Data Collection

Following approval from the Institutional Review Board, a single-site retrospective analysis was conducted at Nationwide Children’s Hospital in accordance with the Helsinki Declaration of 1964 and its later amendments. Patients were identified using the Nationwide Children’s Hospital Heart Center patient database and screened based on identification of their type of VSD as well as whether they underwent surgical closure. Patients less than 21 years of age with a diagnosis of a pVSD who underwent surgical closure between 1 January 2000 and 31 December 2024 were reviewed and their data collected through use of the Research Electronic Data Capture (REDCap 16.0.18) software.

Patients were included if they underwent uncomplicated surgical closure of an isolated pVSD. Patients with additional CHD were excluded, with the exception of secundum atrial septal defects, patent ductus arteriosus, or small muscular VSDs that resolved prior to or at the time of surgery. Patients were categorized as having an uncomplicated surgical closure if their 1-year TTE demonstrated normal chamber sizes and normal biventricular systolic function without complications. The post-repair follow-up timeframe of 1 year was chosen in congruence with current guideline recommendations as well as from prior knowledge that the majority of complications occur within 30 days of repair with 97% of patients demonstrating normalization of left ventricular function 1 year after surgical repair [4,5,6,7]. A complication by TTE was defined as the presence of residual lesions, atrial or ventricular arrhythmias, pulmonary hypertension, a pericardial effusion, or ventricular dysfunction at 1 year post-repair. Additional exclusion criteria included patients who underwent reoperation or had additional surgical procedures either during the initial operation or within the first year following their surgical repair, as these patients would require long-term follow-up. Patients who returned to their referring medical facility or were lost to follow-up within the first year of their surgical repair were excluded as they had insufficient data at 1 year post-repair. Similarly, patients who had not yet reached 1 year post-repair at the time of analysis and thus had insufficient data at 1 year were excluded from the landmark analysis.

### 2.2. Clinical Characteristic and Patient Demographics

Demographic data and clinical characteristics of the population including age at surgical repair and type of surgical closure (patch vs. primary closure) were collected. TTE data was collected on each patient preoperatively and postoperatively with attention to chamber size, atrioventricular valve regurgitation, ventricular function, and the presence of any complications such as ventricular dysfunction, arrhythmias, pulmonary hypertension, outflow tract obstruction, or pericardial effusions. TTE data was noted immediately prior to and following surgical intervention, at 1 year post-repair, during the patients’ follow-up period, and at any point at which a complication occurred. In addition to TTE data, the most recent clinic notes from each patient were reviewed for any clinical concerns or discussion of changes in clinical management.

### 2.3. Study Outcomes

Serial surveillance TTEs in patients who underwent uncomplicated surgical closure of their pVSD and had a normal TTE 1 year post-repair were analyzed to determine if a complication or change in clinical management occurred. If a complication did occur, TTE data was documented for that follow-up period with note of the time interval since surgical repair, and the clinic note was reviewed to determine if any change in clinical management occurred.

### 2.4. Statistical Analysis

Baseline clinical characteristics and TTE data were presented as number and percentages (%) for categorical variables and by means, standard deviations, medians, and interquartile ranges for continuous variables. The Kaplan–Meier estimator was used to demonstrate the percentage of patients who were complication-free at 5 years post-repair using a 12-month landmark approach to account for patients lost to follow-up with the assumption that the majority of immediate post-operative complications, including residual VSD and ventricular dysfunction, should resolve by the 1-year post-repair follow-up TTE [4]. Any complications occurring after this time would be considered a new complication.

## 3. Results

**Figure 1:**
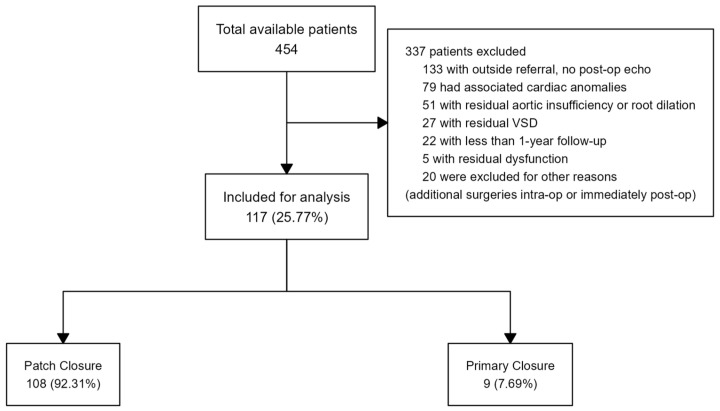
Surgical type and exclusion criteria.

A total of 864 TTEs from 454 unique patients were initially reviewed. Following implementation of exclusion criteria, 117 unique patients remained, contributing to a total of 477 TTEs (Figure 1). Of those, 108 patients underwent patch closure, and 9 patients underwent primary closure of a pVSD with a median age at surgery of 6 months (IQR; 4, 13) (Table 1). In the 117 patients who underwent uncomplicated surgical closure of a pVSD, there were a total of 208 TTEs obtained beyond the 1-year post-repair with a mean of 1.78 TTEs per patient (SD 1.38) and a maximum of 7 TTEs per patient (Table 1). Of the 117 patients included, the mean time for the most recent TTE following surgical repair was 94 months with a median of 82 months (IQR; 35, 133), and the longest follow-up TTE being 266 months post-repair (Table 2). Similarly, the median outpatient follow-up time post-repair was 93 months (IQR: 47, 150). As Table 1 demonstrates, 85 patients (73%) continue to follow with cardiology, 28 patients (24%) were lost to follow-up, and 4 patients (3.4%) were discharged from cardiology (Table 1). There were no patient deaths. As illustrated by the most recent TTE data in Table 2, no patient had a residual VSD or ventricular dilation on their most recent TTE. Additional TTE data and clinical characteristics are depicted in Table 2.

**Figure 2:**
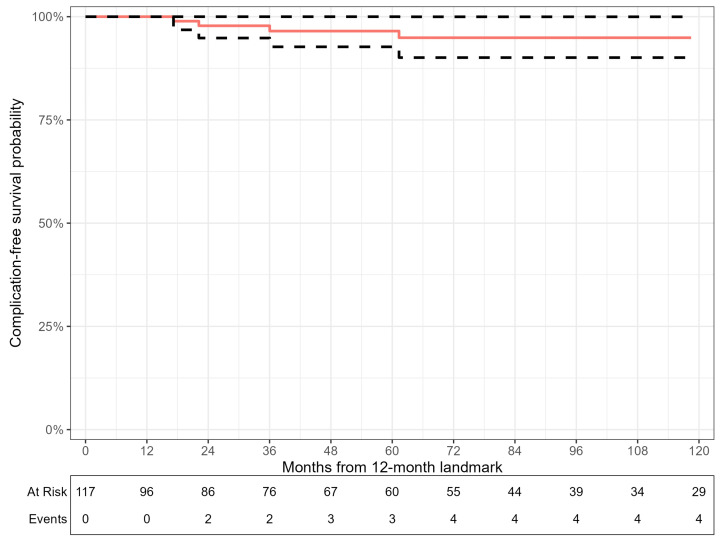
Time to complication. Survival Probability (95% CI).

The Kaplan–Meier curve demonstrated a 97% 5-year complication-free survival with only four patients noted to have a complication after 1 year post-repair (Figure 2). One patient was found to have mild pulmonary vein stenosis (mean gradient 2–3 mmHg), one patient developed ventricular ectopy and left ventricular dysfunction, one patient had a non-obstructive subaortic ridge, and one patient was noted to have a pinhole residual pVSD that was not previously seen on the initial 1-year post-operative TTE (Table 3). Of the total 208 TTEs performed beyond 1 year post-repair, 197 TTEs were obtained in the remaining 113 complication-free patients with no change in clinical management per documentation in their outpatient follow-up visit.

Upon review of the four patient complications, some of the complications were likely incidental and unrelated to a pVSD surgical repair. Additionally, only the patient with ventricular ectopy and resultant dysfunction required medical intervention while other complications resolved or remained stable (Table 3). The patient with mild pulmonary vein stenosis was noted to have increased flow through the pulmonary vein on the 6-month post-repair TTE. Follow-up TTE at 12 months had a normal pulmonary vein Doppler before again demonstrating an abnormal Doppler 29 months after repair. This mild pulmonary vein stenosis is likely an incidental finding and unrelated to the pVSD surgical repair, and the patient continues to have stable pulmonary vein gradients with no cardiac intervention required. As Table 3 demonstrates, the patient who was noted to have ventricular dysfunction 34 months after surgical repair had progressive ventricular ectopy that was first noted within 1 year of surgical repair. This patient required ablation of an ectopic ventricular focus in the right ventricular outflow tract with improvement in the ectopy burden but had ongoing concerns for a left-sided electrical focus as well as late gadolinium enhancement on cardiac magnetic resonance imaging. This patient continued to have ventricular dysfunction and was lost to follow-up (Table 3). The patient who demonstrated a non-obstructive subaortic ridge (peak gradient 4 mmHg) at 48 months post-repair has continued to be asymptomatic with no change in the gradient on follow-up visits nor cardiac intervention required. The final patient with a pinhole residual pVSD had this complication first noted at 73 months post-repair. Upon further review of prior imaging, this residual defect was likely present on previous TTEs, but imaging was difficult due to patient movement. Given the pinhole size and difficult visualization on prior TTEs, this residual pVSD is likely not hemodynamically significant and thus would not be a clinically significant complication that would have resulted in a change in management. Regardless, the residual pinhole pVSD self-resolved on repeat sedated TTE and the patient was subsequently discharged from the cardiology clinic.

## 4. Discussion

Surgical repairs of VSDs have excellent outcomes with low morbidity and mortality [4,5,7,8,9]. North American imaging guidelines currently recommend routine surveillance TTEs at 1 year and then every 2–3 years in patients who underwent surgical repair with no or mild sequela, yet the duration of the TTE follow-up is left to the discretion of the primary cardiologist [6]. In this preliminary analysis evaluating the utility of routine surveillance TTEs following uncomplicated surgical closure of a pVSD, there was a 97% 5-year complication-free survival with only four patients exhibiting complications following a normal TTE at 1 year post-repair. As previously noted, some of these complications were likely incidental and unrelated to a pVSD surgical repair, and only the patient with ventricular ectopy and resultant dysfunction was symptomatic, resulting in a change in medical management. Follow-up TTEs in the remaining patients that had no complications at 1 year post-pVSD repair did not lead to any change in medical or surgical management which further supports the need for re-assessment of the duration of follow-up TTEs in patients who have a normal TTE 1 year after undergoing an uncomplicated pVSD repair.

As previous studies have demonstrated, surgical closures of pVSDs carry low morbidity and mortality [4,5,7,9]. Some common complications that can be seen following surgical repair include residual lesions, atrial or ventricular arrhythmias, aortic valve insufficiency, mitral valve regurgitation, pericardial effusions, or ventricular dysfunction; however, the majority of these complications occur within the early post-operative period following surgical repair [3,4,5,7,9]. Additional complications including transient or persistent complete heart block, pulmonary hypertension, emergent reoperation, neurologic injury, or death were rare, with the majority also occurring in the immediate post-operative period [4,5,7,9]. This data is consistent with previous publications noting the majority of complications detected by TTE occurred <1 year post-operatively and most often within 30 days post-repair [5,7]. Therefore, the recommendation to obtain a TTE within 30 days and then again at 1 year following surgical VSD closure seems justified [6].

The mid and long-term outcomes of pVSD surgical repair are also quite good with minimal morbidity and mortality if there were no underlying risk factors pre-operatively [4,5,7,9]. In this study, there was a 97% 5-year complication-free survival in patients who had no complications at 1 year following an isolated pVSD repair with only four patients noted to have issues beyond the 1-year post-pVSD repair. Furthermore, only one of these complications resulted in a change in clinical management with the other complications being likely incidental and unrelated to the pVSD repair. Of the four patients, the patient with the pinhole residual pVSD likely had the lesion present at 1 year post-repair. Although this residual VSD was not recognized due to difficult image acquisition in an uncooperative patient, it was not clinically nor hemodynamically significant and the patient was ultimately discharged from the cardiology clinic after sedated TTE demonstrated resolution of the residual lesion. The other two patient complications of mild pulmonary vein stenosis and a nonobstructive subaortic ridge were likely not related to the pVSD repair and additionally did not result in a change in medical management. These latter two patients continue to be asymptomatic with no progression of TTE findings and no change in clinical management, and thus are not clinically meaningful complications. Only one patient had significant cardiac complications beyond 1 year following surgical pVSD repair. This patient’s dysfunction was thought to be secondary to a high ventricular ectopy burden leading to ventricular dysfunction; thus, an electrocardiogram (ECG) and ambulatory rhythm monitor would have been the appropriate initial diagnostic tests with a TTE used to assess for secondary findings. This scenario is consistent with the current guidelines to perform a TTE if a “change in clinical status and/or new concerning signs or symptoms” occur [6].

North American imaging guidelines recommend surveillance TTEs at 1 year post-pVSD repair and then every 2–3 years even if there are no or mild sequelae; however, the duration of follow-up TTEs is left at the discretion of the primary cardiologist [6]. North American adult congenital heart disease guidelines recommend TTE every 3–5 years in an asymptomatic patient with a pVSD, though it is unclear if this follow-up recommendation is for patients who underwent pVSD repair or have an unrepaired pVSD [10]. European guidelines state that a cardiology follow-up every 3–5 years post-VSD repair may be reasonable in patients with no concerns or in those with no VSD or only a small VSD; however, there is no mention of TTE use [11]. Though long-term cardiology follow-up should be continued in these patients, this study suggests that routine TTE beyond 1 year after uncomplicated surgical closure of a isolated pVSD is likely not necessary unless there are pre-existing concerns or if new concerns arise. Of the total 208 TTEs performed >1 year post-pVSD repair in the 117 patients meeting the inclusion criteria, only one patient had clinically significant cardiac findings on their TTE. For this patient, the new concern for increased ventricular ectopy would have prompted a follow-up TTE at that time in compliance with current guidelines [6,10,11]. Given the potential costs to families and the healthcare system as well as the lack of clarity among current recommendations regarding the duration of follow-up TTEs, it is important to understand the time interval during which the utility of routine surveillance TTEs in asymptomatic patients or those with mild sequelae following an uncomplicated pVSD repair is warranted.

## 5. Limitations

This study has several limitations which may yield opportunities for future investigations. This is a retrospective study with the inherent limitations of such a design. As a single-site study, the sample size following exclusion criteria was small. Nearly 30 percent of the initial patient population were excluded for lack of TTE data post-repair due to being either a referral from an outside hospital or lost to follow-up. A significant portion of patients were excluded for associated cardiac anomalies or additional surgical interventions at the time of their pVSD repair; thus, these findings are not generalizable to these patients and instead are more representative of the specific patient population that met inclusion criteria. The patient population was relatively homogeneous with a strong pediatric age predilection; thus, these findings should not necessarily be equated to patients who underwent pVSD repair as adults. The longest follow-up time evaluated in this uncomplicated cohort was 22 years; therefore, no specific comment can be made beyond that time frame. Finally, although there are established recommendations for surveillance TTEs during the first year post-repair and beyond, provider practice remained variable with some providers obtaining TTEs more or less frequently than recommended. This practice variation makes it challenging to determine the time at which the majority of patients are most at risk of new complications.

## 6. Conclusions

In patients who underwent uncomplicated surgical closure of an isolated pVSD and had a normal 1-year postoperative TTE, the occurrence of new complications beyond 1 year is rare, with this study demonstrating a 97% 5-year complication-free survival. Unless clinical concerns arise, the utility of routine TTEs following a normal 1-year postoperative TTE may have low utility and should be reassessed in this uncomplicated post-surgical group. Larger multicenter studies with inclusion of residual lesions and other CHDs are needed to determine the utility of routine TTEs as well as the duration of the recommended follow-up after an uncomplicated surgical repair of pVSDs.

## Figures and Tables

**Table 1:** Follow-up outcomes.

Characteristic	Primary Closure N (%) = 9	Patch Closure N (%) = 108	Overall N (%) = 117
Age at Surgery (months)			
Mean (SD)	17 (23)	18 (34)	18 (33)
Median (Q1, Q3)	10 (4, 13)	6 (4, 14)	6 (4, 13)
Min, Max	4, 75	1, 230	1, 230
Total Echoes obtained >1 year post-op			
Mean (SD)	1.33 (1.12)	1.81 (1.40)	1.78 (1.38)
Median (Q1, Q3)	2.00 (0.00, 2.00)	2.00 (1.00, 2.00)	2.00 (1.00, 2.00)
Min, Max	0.00, 3.00	0.00, 7.00	0.00, 7.00
Follow-up Outcome			
Follow with Cardiology	6 (67%)	79 (73%)	85 (73%)
Discharged from Cardiology	1 (11%)	3 (2.8%)	4 (3.4%)
Lost to Follow-up	2 (22%)	26 (24%)	28 (24%)
Deceased	0 (0%)	0 (0%)	0 (0%)

**Table 2:** Surgical and TTE findings.

Characteristic	Pre-op Echo N (%) = 117	Immediate Post-op N (%) = 117	>1 Year Post-op N (%) = 116	Most Recent Post-op N (%) = 117
Time Since Surgery (Months)				
Mean (SD)	−2 (1)	0 (0)	22 (27)	94 (70)
Median (Q1, Q3)	−1 (−2, −1)	0 (0, 0)	13 (6, 22)	83 (35, 135)
Min, Max	−6, 0	0, 1	1, 143	12, 266
VSD				
No	0 (0%)	80 (70%)	115 (100%)	117 (100%)
Yes	117 (100%)	35 (30%)	0 (0%)	0 (0%)
Unknown	0	2	1	0
LA Dilation				
None	19 (16%)	50 (43%)	102 (88%)	115 (98%)
Mild	51 (44%)	59 (50%)	13 (11%)	2 (1.7%)
Moderate	43 (37%)	8 (6.8%)	1 (0.9%)	0 (0%)
Severe	4 (3.4%)	0 (0%)	0 (0%)	0 (0%)
LV Dilation				
None	33 (28%)	83 (71%)	114 (98%)	117 (100%)
Mild	44 (38%)	25 (21%)	2 (1.7%)	0 (0%)
Moderate	34 (29%)	8 (6.8%)	0 (0%)	0 (0%)
Severe	6 (5.1%)	1 (0.9%)	0 (0%)	0 (0%)
Mitral Regurgitation				
None	106 (92%)	101 (88%)	110 (97%)	111 (99%)
Mild	7 (6.1%)	13 (11%)	3 (2.7%)	1 (0.9%)
Moderate	2 (1.7%)	1 (0.9%)	0 (0%)	0 (0%)
Severe	0 (0%)	0 (0%)	0 (0%)	0 (0%)
Unknown	2	2	3	5
RA Dilation				
None	107 (91%)	107 (92%)	114 (98%)	116 (99%)
Mild	10 (8.5%)	9 (7.8%)	2 (1.7%)	1 (0.9%)
Moderate	0 (0%)	0 (0%)	0 (0%)	0 (0%)
Severe	0 (0%)	0 (0%)	0 (0%)	0 (0%)
Unknown	0	1	0	0
RV Dilation				
None	107 (91%)	112 (96%)	116 (100%)	117 (100%)
Mild	10 (8.5%)	5 (4.3%)	0 (0%)	0 (0%)
Moderate	0 (0%)	0 (0%)	0 (0%)	0 (0%)
Severe	0 (0%)	0 (0%)	0 (0%)	0 (0%)
Tricuspid Regurgitation				
None	101 (87%)	91 (78%)	107 (92%)	106 (91%)
Mild	13 (11%)	24 (21%)	9 (7.8%)	11 (9.4%)
Moderate	2 (1.7%)	2 (1.7%)	0 (0%)	0 (0%)
Severe	0 (0%)	0 (0%)	0 (0%)	0 (0%)
Unknown	1	0	0	0
Morbidities/ Complications				
No	113 (97%)	75 (64%)	114 (98%)	113 (97%)
Yes	3 (2.6%)	42 (36%)	2 (1.7%)	4 (3.4%)
Unknown	1	0	0	0
Atrial Arhythmias	0 (0%)	0 (0%)	0 (0%)	0 (0%)
Ventricular Arrhythmias	0 (0%)	0 (0%)	0 (0%)	1 (0.9%)
Pulmonary HTN	0 (0%)	1 (0.9%)	0 (0%)	0 (0%)
Decreased LV Function	0 (0%)	30 (26%)	0 (0%)	1 (0.9%)
LVOTO	0 (0%)	0 (0%)	0 (0%)	1 (0.9%)
RVOTO	0 (0%)	0 (0%)	0 (0%)	0 (0%)
Effusion	0 (0%)	9 (7.7%)	0 (0%)	0 (0%)
Other	3 (2.6%)	3 (2.6%)	2 (1.7%)	2 (1.7%)

HTN = hypertension, LA = left atrial, LV = left ventricle, LVOTO = left ventricular outflow tract obstruction, RA = right atrial, RV = right ventricle, RVOTO = right ventricular outflow tract obstruction, TTE = transthoracic echocardiogram, VSD = ventricular septal defect.

**Table 3:** Complications occurring >1 year post-repair.

	Patient 1	Patient 2	Patient 3	Patient 4
Complications				
Time since surgery	48 months	29 months	73 months	34 months
TTE finding	Non-obstructive subaortic ridge	Mild pulmonary vein stenosis	Pinhole residual VSD	Ventricular ectopy with dysfunction
Symptomatic	No	No	No	Yes
Last Clinic Visit				
Number of TTEs post-repair	2	1	3	3
Last TTE post-repair	85 months	29 months	161 months	80 months
TTE finding	Subaortic ridge still present	Stable RLPV gradient	Resolution of the residual VSD	Persistent ectopy and dysfunction
Change in management	No	No	No	Medication and RVOT ablation
Outcome	Follows with cardiology	Follows with cardiology	Discharged from cardiology	Lost to follow-up

RLPV = right lower pulmonary vein, RVOT = right ventricular outflow tract, TTE = transthoracic echocardiogram, VSD = ventricular septal defect.

## Data Availability

The original contributions presented in this study are included in the article. Further inquiries can be directed to the corresponding author.
